# Communication research to improve engagement with climate change and human health: A review

**DOI:** 10.3389/fpubh.2022.1086858

**Published:** 2023-01-26

**Authors:** Eryn Campbell, Sri Saahitya Uppalapati, John Kotcher, Edward Maibach

**Affiliations:** George Mason University's Center for Climate Change Communication, Fairfax, VA, United States

**Keywords:** health, climate change, air pollution, fossil fuels, climate change communication, climate solutions

## Abstract

Because of the world's dependence on fossil fuels, climate change and air pollution are profoundly harming both human and planetary health. Fortunately, climate solutions are also health solutions, and they present both local and global opportunities to foster cleaner, healthier, and safer communities. In this review, we briefly discuss the human health harms of climate change, climate and health solutions, and provide a thorough synthesis of social science research on climate and health communication. Through our review, we found that social science research provides an evidence-based foundation for messaging strategies that can build public and political will for climate and health solutions. Specifically, messages that convey the health harms of climate change and highlight the health benefits of climate solutions may be especially effective in building this public and political will. We also found that health professionals are trusted sources of information about climate change, and many have shown interest in engaging with the public and policymakers about the health relevance of climate change and clean energy. Together, the alignment between message strategies and the interest of highly trusted messengers strongly suggests the potential of health students and health professionals to create the conditions necessary to address climate change as a public health imperative. Therefore, our review serves as a resource for those interested in communicating about climate change and health and suggests that social scientists can continue to support practitioners with research and advice on the most effective communication strategies.

## 1. Introduction

Climate change and air pollution—both of which are primarily caused by the world's reliance on fossil fuels (i.e., coal, oil, and natural gas)—are arguably among the leading causes of morbidity and mortality worldwide, and the magnitude of these linked problems is growing rapidly (1, 2). Therefore, fossil fuel use is the world's most pressing public health problem, and decarbonizing communities and nations is one of the world's most promising public health opportunities.

Social science research is playing—and will continue to play—an important role in addressing these challenges. To demonstrate this, we begin this review by providing a brief overview of the public health emergency that is being caused by climate change and fossil fuel use and the solutions that have the potential to quickly improve public health while also helping to stabilize the world's climate over time. After providing a description of the problem and the potential solutions and their benefits, we synthesize the social science research on how to educate the public and policymakers about the human health relevance of climate change and build public support for the policies necessary to protect human and planetary health. By doing so, we summarize an important and growing body of work, providing a resource for those interested in communicating about climate change and health and a foundation for future research.

## 2. The health harms of fossil fuel use, climate change, and air pollution

By adding large amounts of heat-trapping pollution—like carbon dioxide and methane—into the Earth's atmosphere during the twentieth and twenty-first centuries, the fossil fuel industry has become the primary driver of poor air quality and climate change globally (1, 2). Together, climate change and air pollution from burning fossil fuels are already harming both human and planetary health on an unprecedented scale, signaling a major public health concern.

**Figure 1:**
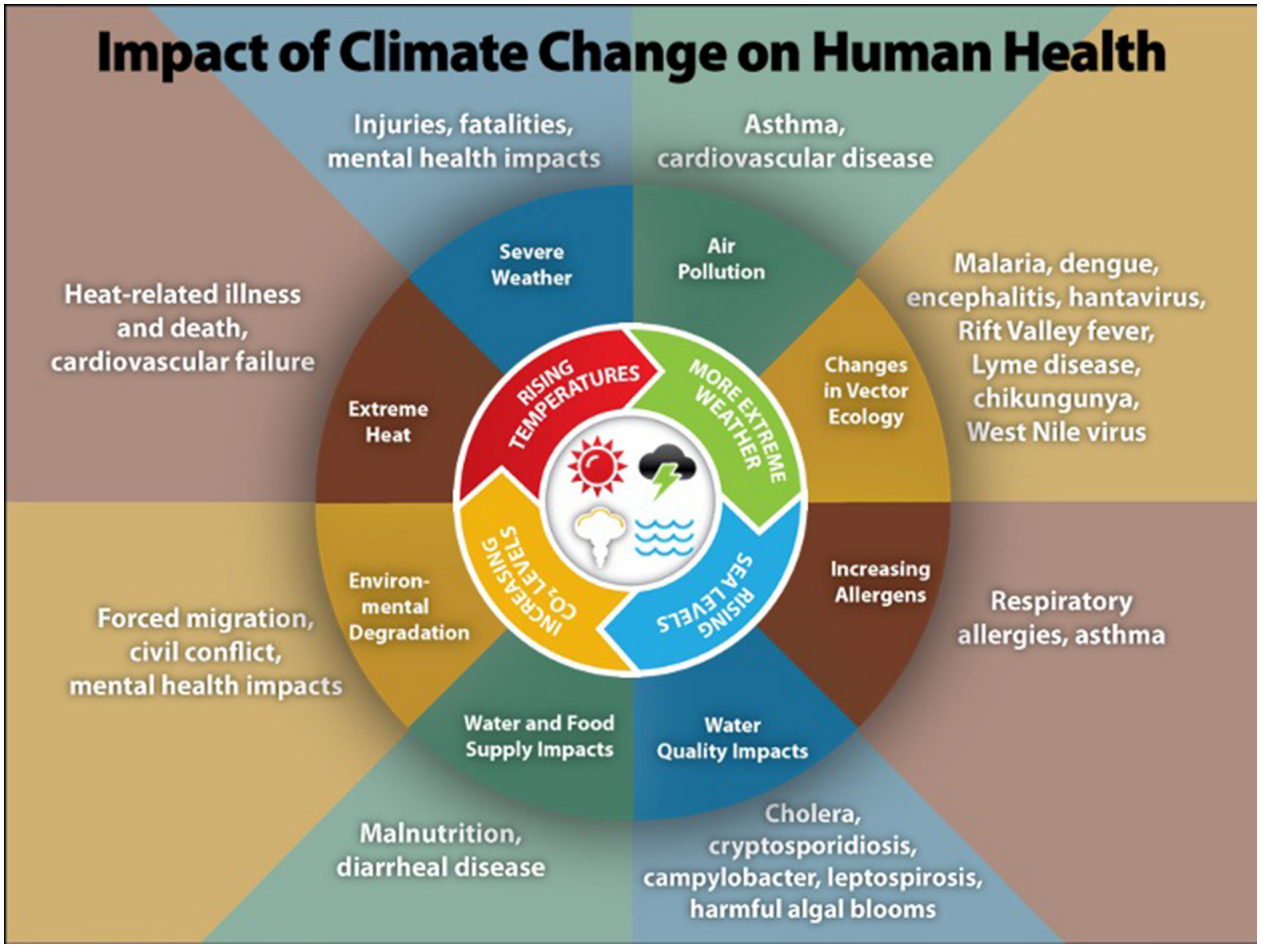
Illustration of the most significant climate change impacts, their effect on exposures, and the subsequent health outcomes that can result from these changes in exposures [from the Center for Disease Control (4)].

Figure 1 summarizes the ways climate change can harm human health, including increases in heat-related illnesses and deaths; vector-, water-, and food-borne diseases; respiratory diseases due to reduced outdoor air quality; food insecurity and malnutrition; and direct and indirect physical and mental harm from extreme weather events and wildfires (3). The geographic range, frequency, and severity of these impacts are projected to continue to grow if preventive actions are not taken (3). Importantly, these health harms disproportionately affect people in low-income and minority communities, exacerbating existing health disparities and inequities like access to clean air and water (3).

According to the latest reports by the Fourth U.S. National Climate Assessment (NCA4), the Intergovernmental Panel on Climate Change (IPCC), and the 2022 Lancet Countdown on Health and Climate Change, climate-related health impacts are increasing in the United States and worldwide (3, 5, 6). The impacts of summer heat waves are one indicator of this increase. From 2000 to 2021, people were exposed to an average summer temperature of half a degree Celsius higher than the average from 1986 to 2005; such exposure can lead to illness or death and restricts people's ability to work or exercise (6). Furthermore, heat-related deaths among those 65 years and older increased by 68% from 2000–2004 to 2017–2021 (6). By 2100, the percentage of the global population exposed to deadly heat stress is projected to increase from 30 to 48–74%, depending on emission scenarios and population distribution (7). Furthermore, according to the IPCC AR6 risk report, other health risks—such as water- and vector-borne diseases—will become more severe at both global and regional levels with increased warming and vulnerability (3).

Poor air quality is one of the most harmful health impacts of climate change. One indicator of this is wildfire exposure, as climate change can lead to poor air quality through increases in wildfire smoke. From 2001–2004 to 2018–2021, the number of days of human exposure to very- or extremely high fire danger increased in 61% of countries, meaning more people were exposed to poor air quality from wildfire smoke, suffered the loss of infrastructure, and may have experienced lasting mental health impacts (6).

Furthermore, uncontrolled fossil fuel use produces air pollution which in turn drives climate change. Together, air pollution and climate change are one of the leading causes of morbidity and mortality worldwide. From 2012 to 2018, air pollution from fossil fuels was estimated to be responsible for 8.7 million premature deaths per year globally (8). In the United States, over 40% of the population (more than 137 million people) live in areas with unhealthy levels of particulate pollution or ozone (9). Children are especially vulnerable to the health harms of air pollution. Prenatal and early childhood exposure to air pollution caused by the burning of fossil fuels has been linked to impacts on children's brain development, including delayed development, reduced IQ, symptoms of anxiety and depression, inattention, increased risk of autism, and premature and low-weight births that may increase the risk of neurological disorders (10). This dire public health problem can be addressed by phasing out fossil fuel use, which would reduce outdoor air pollution and prevent the loss of up to 3.61 million lives per year (11).

While the greatest cost posed by fossil fuel use is on people's health and wellbeing, there are also significant economic costs associated with the health impacts of both climate change and air pollution caused by the burning of fossil fuels. Already, the combined health costs attributed to climate change and air pollution amount to over $800 billion per year just in the United States (12). Globally, health damages as a result of exposure to air pollution alone amount to $8.1 trillion (13).

The wide-ranging health impacts of fossil fuel use—and the resulting air pollution and climate change—on human health demonstrate that this is a complex public health issue that will continue to worsen if countries do not phase out the use of fossil fuels. Addressing this issue will require viable, accessible, and cost-effective climate solutions that mitigate the drivers of these harms while also improving human health and advancing equity.

## 3. Climate solutions are health solutions

Most climate solutions and adaptation measures that have links to health can be categorized into three broad types: (1) solutions that reduce the emission of heat-trapping pollution and transition to clean energy, (2) solutions that reduce the amount of carbon pollution in the atmosphere, and (3) solutions and adaptations that enhance community preparedness (14). Many of the actions in each of these categories also produce health benefits and, if done right, equity benefits (15). Therefore, climate solutions have “co-benefits” that can quickly improve public health and wellbeing while also helping to stabilize the climate (16–19). Table 1 provides examples of the types of climate solutions and adaptation measures and their associated climate and health benefits.

**Table 1:** Summary of example climate solutions and adaptation measures with related climate and health benefits.

**Solution type**	**Example solutions**	**Benefits for climate and health**
Type 1	• Transitioning to renewable energy • Electric heat pumps and induction stoves • Electrifying transportation • Expanding public transportation • Infrastructure for pedestrian- and cyclist-friendly communities • Plant based diets • Increasing access to family planning	• Reduce heat-trapping emissions from household fossil fuel use, vehicle transport, energy-intensive livestock farming and consumption of energy resources • Clean air and water • Reduce air pollution • Increase physical activity • Decrease stress and improve mental health • Access to reproductive health care • Promote gender equality
Type 2	• Forest restoration • Improved soil management • Greening urban and suburban spaces	• Reduce atmospheric heat-trapping pollution *via* sequestration in plant tissues and soils • Reduce flooding and resulting mold • Reduce urban heat islands • Reduce pesticide exposure • Improve mental health • Increase food security
Type 3	• Community cooling centers • Improved control measures for vector-borne diseases • Access to mental health resources and therapists	• Increase community preparedness for climate impacts • Limit exposure to extreme heat • Limit spread of disease • Improve mental health

Type 1 solutions that reduce emissions of heat-trapping pollution—and thereby reduce air and water pollution and improve human health—include rapidly transitioning away from fossil fuels to clean, reliable, and renewable energy sources [e.g., solar, wind, and geothermal; (6, 15)]; heating and cooling buildings and water with electricity-powered heat pumps and geothermal HVACs (20, 21); cooking with electricity-powered induction stoves (22); and electrifying all possible modes of transportation [cars, trucks, and buses; (15, 23)]. Developing pedestrian- and cyclist-friendly communities and effective, affordable public transit options are additional solutions to reduce air pollution, limit climate change, increase physical activity, reduce obesity, and improve mental health (15, 24, 25). Other measures can result in emission reductions while simultaneously addressing broader societal and health needs (21). For example, promoting plant-based diets and reducing food waste can also reduce emissions and enhance human health (26). Similarly, increasing access to family planning resources and educating girls can help slow future population growth and emission rates while also improving gender equality, access to education, and reproductive healthcare (19, 21).

At present, the primary Type 2 solutions to reduce carbon pollution in the atmosphere are nature-based, although technology-based carbon removal is an area of active research and development. Nature-based solutions include forest restoration, improved soil management practices for agriculture, greening urban and suburban spaces, and composting food waste (21). These actions also benefit human health by reducing urban heat islands, reducing flooding and associated health risks (e.g., mold), reducing exposure to pesticides and other agricultural chemicals, and improving mental health (15).

Finally, Type 3 solutions encompass adaptations that can enhance community resilience to the harmful impacts of climate change, often reinforcing Type 1 and Type 2 solutions. Examples of public health resilience measures include establishing community cooling and clean air centers to limit exposure to dangerous heat and air pollution (27, 28); improving control measures for vector-borne diseases (6, 29); and providing counseling to help people cope with mental health impacts of climate change, including climate anxiety and depression and post-traumatic stress disorder resulting from exposure to extreme weather events (30).

Put simply, *climate solutions are health solutions*, and they present local, national, and global opportunities to foster cleaner, healthier, and safer communities, reduce morbidity and premature mortality, and lower health costs (11). When designed and implemented wisely, climate solutions can also help redress systemic and social inequalities and ensure fair and equitable access to the social and environmental determinants of health, which include clean energy, air, and water; affordable, safe, and nutritious food; a safe and secure neighborhood with access to green spaces; and economic security.

Building enduring public and political will for climate and health solutions may therefore be the most important—and promising—public health objective for the next several decades. Health professionals have long intuited that acknowledging and promoting the human health benefits of climate solutions as “co-benefits” of climate action would help advance this objective (16–19). Social science research conducted over the past decade has confirmed this intuition and refined it.

## 4. Social science research on messages that build public and political will for climate and health solutions

Public understanding of the health relevance of climate change seems limited, although it appears to be growing. As recently as 2014, about six in 10 (61%) Americans had given “little or no thought” to how global warming might impact human health, and relatively few could name a single way in which climate change harms health or whose health is most likely to be harmed (31). A 2018 review of peer-reviewed studies on public awareness of the health relevance of climate change worldwide yielded similar findings (32). Between 2014 and 2020, however, Americans' understanding of the health consequences of climate change grew substantially (33, 34).

Social science research has shown that communicating the health relevance of climate change can increase public engagement with the issue (35, 36). Most fundamentally, presenting information about how climate change harms health and whose health is most likely to be harmed can increase people's concern about and engagement with the issue (37, 38). Moreover, providing information about the health benefits of climate solutions can enhance people's intentions to advocate for such solutions (39). Certain health benefits of climate solutions are more compelling than others, with messages about the health benefits of clean energy and improved community design being the most compelling (39). Including a call to action for climate solutions advocacy that demonstrates how many others are engaging in advocacy (i.e., a social norm) can further enhance the effectiveness of advocacy appeals (39). Among certain vulnerable populations (e.g., low income, less educated, and those with preexisting health conditions), communication that makes the connection between climate and health has also been shown to increase the understanding of the issue and intention to take action (38). Finally, including information about the bad-faith actors in the climate discussion—like the CEOs of fossil fuel companies and politicians working against climate solutions—can also increase the effectiveness of climate and health messages by enhancing emotional engagement with the issue, policy support, and advocacy intentions (40).

A multinational study showed that providing health-framed information about climate change can significantly increase public support for climate mitigation policies, including among people who are not concerned about climate change *per se* (41). This finding—that health-framed climate messaging is effective with people who are not necessarily concerned about climate change—has been demonstrated in other studies as well (35–37, 39), suggesting that climate/health communication may be an important strategy for reducing political polarization about the value of climate solutions.

Similarly, messages that focus on the health harms of fossil fuels and air pollution have also been shown to increase public understanding of these issues, support for clean energy, and intentions to advocate for solutions (39, 42–44). In communication research focused specifically on climate change, messages about poor air quality are the most compelling form of climate change-related health harm (37, 39). Furthermore, one study suggested that air pollution messages may be more effective than climate change messages in building support for clean energy policies (44). Moreover, messages about the neurological harms of air pollution on babies (including before birth) and children are of particular concern to people (42). Other research shows that presenting information about policies aimed at reducing air pollution, as opposed to those aimed at addressing climate change outright, may increase Republican support for such policies (45). Health-oriented messages may be a more compelling reason to reduce fossil fuel use among conservatives compared to climate-oriented messages, which are more compelling among liberals (46).

Among Americans, people's understanding of climate change as a health issue is associated with their broader climate attitudes and beliefs (34). Prior research with Americans identified a spectrum of six distinct audiences, also known as Global Warming's Six Americas,^1^ ranging from the Alarmed (i.e., those who are very worried and engaged with climate change) to the Dismissive (i.e., those who do not believe in the reality of climate change and rather likely consider it a hoax). When looking at how Americans' understanding of climate and health changed over the period from 2014 to 2020, the understanding increased among four of the six segments—the Alarmed, Concerned, Cautious, and Disengaged—while little or no change occurred among the two most climate-skeptical groups, the Doubtful and Dismissive (34).

## 5. Social science research on climate and health messengers

Well-crafted messages can only be successful if delivered by trusted sources who are effective communicators. In April 2022, nearly seven in 10 (69%) U.S. voters said they trust their primary care doctor as a source of information about global warming; relative to most other sources, Republicans were especially likely to trust their primary care doctor as a source of global warming information (47). This role as a trusted communicator may allow health professionals to communicate effectively about topics that otherwise may be perceived as controversial. For instance, one study demonstrated that calling-out opponents of climate change did not diminish health professionals' credibility as a source of information about climate change; in fact, it led to greater trust in health professionals (40).

In addition to being trusted, health professionals also have many relevant skills and knowledge as well as many opportunities to be effective communicators on climate and health (31). Because of this, health professionals and health organizations are increasingly being called upon to educate and engage the public and push for climate-friendly policies and actions (48, 49).

Internationally, many health professionals are concerned about climate and health and would like to see strong climate policies enacted. Many, however, feel they lack the knowledge, time, or peer support to effectively educate the public and policymakers about the issues (50–54). These research insights help design strategies to educate and activate health professionals as climate advocates.

In a 2020 multinational survey of health professionals, most participants expressed the view that health professionals have a responsibility to bring the health impacts of climate change to the attention of the public (86%) and policymakers (90%), and about one-fourth (26%) were willing to participate in a global advocacy campaign to encourage world leaders to implement climate and health solutions (50). Interviews with hospital employees also demonstrated that health professionals are receptive to climate and health information and may be willing to advocate for solutions in their hospitals (55). Other studies asked members of specific medical societies—including the American Thoracic Society, the National Medical Association, and the American Academy of Allergy Asthma and Immunology—similar questions and found similar results, with majorities of members indicating that health professionals should be playing a role in responding to climate change and educating the public (52–54). Feeling a sense of professional responsibility is related to health professionals' willingness to advocate for climate and health solutions (56).

While research shows that many health professionals are ready and willing to act as climate and health communicators and advocates, the barriers they face must be addressed to translate this willingness into action. Luong et al. (51) separated these barriers into three categories: (1) skills and abilities (i.e., knowledge, communication ability, and resource access); (2) environmental constraints (i.e., time constraints and leadership support); and (3) intentions (i.e., perceptions of advocacy's risks/benefits, effectiveness, and social acceptability). Some ways to address these barriers include continuing professional education and communication training; providing resources such as patient education materials and policy statements; demonstrating how to make healthcare workplaces climate-friendly; promoting workplace policies and professional cultures that are supportive of advocacy; and highlighting successful advocacy efforts and outcomes (50, 51).

## 6. Limitations and future research

There are several limitations of our review and areas for future research. First, there is currently not enough research to conduct a quantitative meta-analysis of this literature. Second, our overviews of the health harms of climate change and climate and health solutions are not comprehensive, as their purpose was to set the stage for the larger discussion of social science research on climate and health communication. Other resources can provide much more detail on these points [see IPCC (3), USGCRP (5), Romanello et al. (6)]. Third, much of the research to date has been conducted in the United States, and therefore, our review is U.S.-centric. Future research should seek to explore public perceptions of climate change as a human health issue and test the effectiveness of different climate and health messaging strategies in other countries. Fourth, there is minimal research focused on effective communication with the populations most vulnerable to the health impacts of climate change; this gap should be remedied to better understand how to support these communities. Finally, much of the research on health professionals as climate and health communicators is based solely on cross-sectional survey data. Future research should investigate messaging and behavior change strategies that can effectively engage health professionals in public communication and advocacy for climate and health solutions.

## 7. Conclusion

Because fossil fuel use, air pollution, and climate change are causing profound public health harm and changes in public policy are needed to prevent these harms from escalating, building public and political will for equitable climate and health solutions is a public health imperative. Current research demonstrates avenues for effective communication strategies to engage the public with climate and health topics, though it is important to note that simply providing the public with information does not directly bring about social and societal changes. Public will can help drive political will by making support for pro-climate policies and actions visible to those in positions to effect change. But, for substantive actions to be born out of this public will, trusted stakeholders (including health professionals, scientists, and others) must engage in productive collaborations with those in positions of power—including policymakers and other government officials, industries, corporations, and the news media—to translate public support into effective policies and actions.

While the communication strategies and messages outlined in this review are a starting point, future research should continue to explore (1) how to activate and support health professionals in their climate communication and advocacy efforts, including refining message strategies that have the most potential to create enduring public and political will for policies that protect human health and our climate and (2) how to facilitate the collaborations necessary for large-scale action. Social science research will continue to play an important role in addressing this imperative, and we encourage social science students and social scientists to join this effort. We also encourage health students, health professionals, and others working to protect human health to use their trusted voices to educate the public and policymakers about the health relevance of climate change and the health opportunities inherent in climate solutions. Now is the time to act together in defense of human health and the climate on which we all depend.

## Author contributions

EC: project administration, writing—original draft preparation, and writing—reviewing and editing. SU, JK, and EM: writing—reviewing and editing. All authors contributed to the article and approved the submitted version.

## Footnotes

^1^For more information on Global Warming's Six Americas, see: https://climatecommunication.yale.edu/about/projects/global-warmings-six-americas/.
